# Prognostic impact of a tumor-infiltrating lymphocyte subtype in triple negative cancer of the breast

**DOI:** 10.1007/s12282-020-01084-1

**Published:** 2020-03-28

**Authors:** Tsengelmaa Jamiyan, Hajime Kuroda, Rin Yamaguchi, Yoshimasa Nakazato, Shuhei Noda, Masato Onozaki, Akihito Abe, Mitsuhiro Hayashi

**Affiliations:** 1 Department of Diagnostic Pathology, Dokkyo Medical University, 880 Kitakobayashi, Mibu, Tochigi 321-0293 Japan; 2 Department of Pathology & Laboratory Medicine, Kurume University Medical Center, Kurume, Japan; 3 Breast Center, Dokkyo Medical University, Dokkyo, Japan; 4 Department of Surgery II, Dokkyo Medical University, Dokkyo, Japan; 5 Department of Pathology and Forensic Medicine, Mongolian National University of Medical Sciences, Ulan Bator, Mongolia

**Keywords:** Breast, Triple negative cancer, CD4, CD8, FOXP3

## Abstract

**Background:**

Tumor-infiltrating lymphocytes (TILs) have recently been reported as an important factor in the tumor microenvironment and influence the growth and progression of cancer. However, the relationship between immune cell subpopulations, such as CD4+, CD8+, and FOXP3+, in breast cancer, especially in triple negative carcinoma (TNC), remains unclear.

**Methods:**

The subjects were 107 patients with TNC that were surgically resected at Dokkyo Medical University Hospital between 2006 and 2018. The expression of CD4+, CD8+, and FOXP3+ was evaluated in TILs and expressed as the numbers of positive cells.

**Results:**

Univariate analysis revealed that the TILs were not prognostically significant. In multivariate analyses, increased infiltration of intratumoral (i) CD4+ TILs was found to have a good prognosis in relapse-free survival (RFS). In contrast, a high stromal CD8+ TILs level was found to be a favorable prognostic factor in RFS (*p* = 0.038) and overall survival (OS) (*p* = 0.046). A low sFOXP3 + TILs level was significantly associated with favorable RFS (*p* < 0.001) and OS (*p* = 0.029).

**Conclusions:**

The present study demonstrated no difference in TILs and survival in TNC. However, there was a significant correlation in prognosis with levels of iCD4+, sCD8+, and sFOXP3 + TILs in TNC. The difference in TNC clinical outcome may be due to the subtype of the infiltrating TILs.

## Introduction

Gene expression profiling studies have divided invasive breast cancer into several major subtypes [1]. The so-called ‘triple negative carcinoma’ (TNC) is characterized by a lack of expression of the estrogen receptor (ER) and progesterone receptor (PgR), and absence of human epidermal growth factor receptor 2 (HER2) protein overexpression; this type is known to have a poor prognosis [2, 3]. Tumor-infiltrating lymphocytes (TILs) have recently been reported as an important factor in the tumor microenvironment and influence the growth and progression of cancer.

The majority of TILs in cancer are of the T-cell phenotype, which includes CD4+ (helper cells) and CD8+ (cytotoxic cells) lymphocytes. CD4 + T lymphocytes are important for priming tumor-specific CD8 + TILs as well as for the secondary expansion and memory of CD8 + TILs [4]. Furthermore, many immunohistochemical studies have concluded that CD8 + TILs have antitumor activity as evaluated by the favorable prognosis in colorectal [5], ovarian [6], esophageal [7], renal [8], lung [9], and pancreatic [10] tumors. However, the impact of CD8 + TILs in breast cancer is controversial. Previous breast cancer studies have reported that marked infiltration of CD8 + TILs is associated with good prognosis, while several studies have found a negative correlation or no correlation with prognosis [11–14]. Therefore, the assumption that lymphocyte infiltration promotes or prevents cancer cannot be confirmed without clarifying which immune cell phenotype is involved.

The role of CD4 + TILs in immune activity has been reported in many cancer patients. However, the discovery of regulatory T cells (Treg) has markedly changed conventional speculation regarding the role of CD4 + T lymphocytes in anti-tumor immunity.

Forkhead box protein 3 (FOXP3) plays a critical role in the generation of immune-suppressive CD4 + Tregs, and this leads to immune tolerance of CD8+ killer cells [15]. Excess FOXP3 expression leads to Treg proliferation and severe immunodeficiency, whereas lack of FOXP3 results in immune system activation and aggressive lymphoproliferation. Furthermore, FOXP3 is involved in immune escape mechanisms and both poor survival and improved survival in breast cancer have been reported [15–17]. There have been several reports on TILs in breast cancer [18, 19]. However, the relationship between immune cells subpopulations, such as CD4+, CD8+ and FOXP3+, in breast cancer, especially in TNC, remains unclear. We herein discuss the clinicopathological features and possible roles of immune cells in TNC.

## Materials and methods

### Patients

The subjects were 107 patients with TNC, which was surgically resected at Dokkyo Medical University Hospital between 2006 and 2018. Patients’ clinical information was retrieved from institutional medical records. Clinical outcome was also documented. For each case, all available hematoxylin and eosin-stained whole-tissue sections were reviewed to confirm the diagnosis of mammary disease with no knowledge of prior histological results or clinical outcomes. The present study was approved by the Ethics Committees of Dokkyo Medical University (Tochigi, Japan; registration number 28009).

### Immunohistochemistry (IHC)

**Fig. 1:**
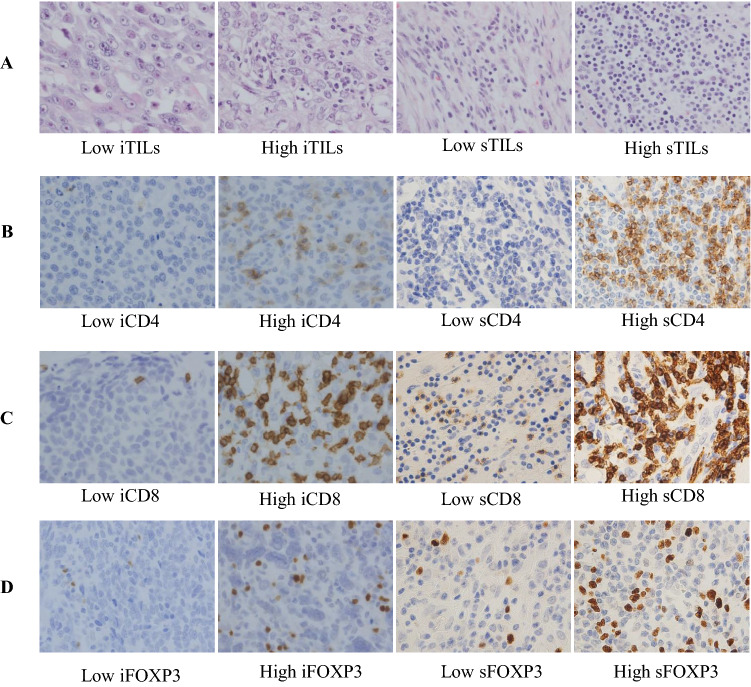
Triple-negative carcinoma of the breast. **a** Representative H&E staining images of iTILs and sTILs. **b**–**d** Representative images of immunohistochemical staining of low and high CD4+, CD8+ and FOXP3 + TILs infiltration densities in intratumoral and stromal areas. **b** CD4 + TILs, **c** CD8 + TILs, and **d** FOXP3 + TILs. Original magnification: ×400

Surgical sections were immunostained for ER (clone SP1, Novocastra (Leica), prediluted, nuclear), PgR (clone 1E2, Novocastra (Leica), prediluted, nuclear), HER2 (clone 4B5, Roche (VENTANA), prediluted, membranous), CD4 (CD4, clone 1F6, Novocastra (Leica), 1:40), CD8 (CD8, clone 4B11, Novocastra (Leica), prediluted) and FOXP3 (FOXP3, clone 236A/E7, abcam, 1:50). Counterstaining was performed with hematoxylin. ER and PgR status were considered positive if any positive cells were detected within the tumor. HER2 status was assessed according to the guidelines defined by the American Society of Clinical Oncology/College of American Pathologists [20]. We estimated the TILs on hematoxylin and eosin (H&E) stained sections according to the criteria proposed by the International Immuno-Oncology Biomarkers Working Group [21]. Lymphocytes in contact with or within the tumor epithelium were defined as intratumoral (i), whereas lymphocytes in the interstitial space or in the stromal areas were defined as stromal (s). TILs were defined as all mononuclear cells, including lymphocytes, within the stromal area, and excluded necrosis, crush artifacts, regressive hyalinization, as well as granulocytes and other polymorphonuclear leukocytes (Fig. 1). TILs levels were categorized as high (≥ 30%) and low (< 30%) adopting previously validated cut-offs [22]. The expressions of CD4, CD8, and FOXP3 were evaluated in TILs and expressed as the numbers of positive cells counted in each case at ×400 magnification (×40 objective) (Fig. 1). For statistical analyses, the number of positive cells was divided into lower and higher groups based on cut-off points according to the median. As a result, the cut-off for iCD4+ was 3, sCD4+ was 54, iCD8+ was 7, sCD8+ was 43, iFOXP3+ was 3, and sFOXP3+ was 32. All sections were evaluated by two pathologists (TJ and HK) who had no previous knowledge of the patients’ clinical information, and the results were averaged.

### Statistical analysis

The associations between CD4+, CD8+, and FOXP3 + TILs and clinicopathological variables were examined by *x*^2^*-*test. Relapse-free survival (RFS) was defined as the time from surgery to recurrence, including metastatic disease. Overall survival (OS) was determined from the date of surgery to the date of death by cancer or to the date of the last follow-up. For significance testing in Kaplan–Meier survival analysis, we used the log-rank test. The hazard ratios (HRs) and 95% confident intervals (CIs) were calculated by Cox proportional hazard models. Multivariate Cox regression analysis including all potential variables that were significantly associated with survival in each univariate analysis was performed. All statistical tests were considered significant at the *p* < 0.05 level. Statistical analysis was performed using IBM SPSS Statistics 25 (IBM, Armonk, NY, United States).

## Results

Clinicopathological findings and expression of immune markers (CD4 + , CD8 + , FOXP3 +) are summarized in Tables 1 and 2, respectively. The patient ages ranged from 28 to 89 years, with a mean of 58.9 years. Tumor size ranged from 0.3 to 10.0 cm; 64.5% were ≤ 2.0 cm and 35.5% were > 2.0 cm in diameter. They presented with histological high grade (76/107) and lymph node metastasis (30/107). Recurrence occurred in 19 (19.2%) of 99 patients, and cancer-associated death occurred in 12 (12.1%) of 99 patients. Median follow-up for the assessment of RFS was 42.13 (0–120) months and that for overall survival was 43.86 (0–120) months. The high expression of iCD4 + TILs was associated with larger tumor size (*p* = 0.034) and higher histological grade (*p* = 0.006). Lymph node status (*p* = 0.036, *p* = 0.05) and expression of CD8 + TILs in both *i* and *s* areas had a significant correlation in the *x*^2^*-*test.

**Table 1:** Clinicopathological factors of triple-negative cancer (TNC) and the status of intratumoral CD4, CD8, and FOXP3 (*N* = 107)

Clinicopathological parameter	Total no. of cases	iCD4			iCD8			iFOXP3			iCD4/CD8			iCD8/FOXP3			iFOXP3/CD4		
		Low	High	*P* value*	Low	High	*P* value*	Low	High	*P* value*	Low	High	*P* value*	Low	High	*P* value*	Low	High	*P* value*
Age (years)				*0.908*			*0.925*			*0.752*			*0.503*			*0.778*			*0.916*
< 60	52	28	24		*26*	*26*		*29*	*23*		27	25		26	26		27	25	
≥ 60	55	29	26		*28*	*27*		*29*	*26*		25	30		26	29		28	27	
Tumor size (cm)				0.034*			*0.199*			*0.292*			0.027*			*0.850*			0.025*
≤ 2	69	42	27		*38*	*31*		*40*	*29*		39	30		34	35		41	28	
> 2	38	15	23		*16*	*22*		*18*	*20*		13	25		18	20		14	24	
Histological grade				0.006*			*0.880*			*0.073*			*0.093*			*0.191*			*0.083*
I and II	31	23	8		*16*	*15*		*21*	*10*		19	12		12	19		20	11	
III	76	34	42		*38*	*38*		*37*	*39*		33	43		40	36		35	41	
Lymph node status				*0.093*			0.036*			*0.496*			*0.876*			0.033*			*0.094*
Absent	65	30	35		*27*	*38*		*33*	*32*		32	33		25	40		31	34	
Present	30	21	9		*21*	*9*		*19*	*11*		15	15		19	11		20	10	
N/A	12	6	6					6	6					8	4		4	8	
iTILs				*0.633*			0.001*			0.003*			*0.386*			*0.146*			*0.386*
Low	54	30	24		36	18		37	17		24	30		30	24		30	24	
High	53	27	26		18	35		21	32		28	25		22	31		25	28	
sTILs				*0.26*			*0.148*			*0.076*			*0.396*			*0.275*			*0.216*
Low	58	28	30		*33*	*25*		*36*	*22*		26	32		31	27		33	25	
High	49	29	20		21	28		22	27		26	23		21	28		22	27	
Relapse-free survival				*0.536*			*0.066*			*0.852*			*0.837*			*0.361*			*0.837*
No recurrence	80	40	40		36	44		44	36		40	40		37	43		40	40	
Recurrence	19	11	8		13	6		10	9		9	10		11	8		10	9	
Overall survival				0.019*			*0.059*			*0.129*			*0.059*			*0.466*			*0.07*
Alive	87	41	46		*40*	*47*		*45*	*42*		40	47		41	46		41	46	
Died	12	10	2		9	3		9	3		9	3		7	5		9	3	

*FOXP3* Forkhead box P3, *TNC* triple-negative cancer, *N/A* not applicable,* i* intratumoral,* s* stromal, *TILs* Tumor-infiltrating lymphocytes

*P value is significant

*χ2 test

**Table 2:** Clinicopathological factors of triple-negative cancer (TNC) and the status of stromal CD4, CD8, and FOXP3 (*N* = 107)

Clinicopathological parameter	Total no. of cases	sCD4			sCD8			sFOXP3			sCD4/CD8			sCD8/FOXP3			sFOXP3/CD4		
		Low	High	*P* value*	Low	High	*P* value*	Low	High	*P* value*	Low	High	*P* value*	Low	High	*P* value*	Low	High	*P* value*
Age (years)				*0.379*			*0.702*			*0.631*			*0.379*			*0.925*			
< 60	52	23	29		18	34		25	27		29	23		26	26		25	27	*0.631*
≥ 60	55	29	26		21	34		29	26		26	29		27	28		29	26	
Tumor size (cm)				*0.830*			*0.186*			*0.199*			*0.553*			*0.943*			*0.379*
≤ 2	69	33	36		22	47		38	31		34	35		34	35		37	32	
> 2	38	19	19		17	21		16	22		21	17		19	19		17	21	
Histological grade				*0.211*			*0.134*			*0.564*			*0.409*			*0.26*			*0.483*
I and II	31	18	13		14	13		17	14		14	17		18	13		14	17	
III	76	34	42		25	55		37	39		41	35		35	41		40	36	
Lymph node status				*0.089*			0.05*			*0.794*			*0.566*			0.034*			*0.789*
Absent	65	37	28		18	47		34	31		36	29		26	39		33	32	
Present	30	10	20		16	14		15	15		14	16		18	12		16	14	
N/A	12	6	6		5	7		5	7		5	7		9	3		5	7	
iTILs				*0.770*			*0.597*			*0.772*			*0.925*			*0.628*			*0.922*
Low	54	27	27		21	33		28	26		28	26		28	26		27	27	
High	53	25	28		18	35		26	27		27	26		25	28		27	26	
sTILs				*0.942*			*0.249*			*0.066*			*0.752*			*0.916*			*0.622*
Low	58	28	30		24	34		34	24		29	29		29	29		28	30	
High	49	24	25		15	34		20	29		26	23		24	25		26	23	
Relapse-free survival				0.013*			< 0.001*			< 0.001*			0.006*			< 0.001*			0.001*
No recurrence	80	42	38		23	57		48	32		45	35		32	48		48	32	
Recurrence	19	4	15		14	5		2	17		4	15		19	0		3	16	
Overall survival				*0.331*			0.004*			0.012*			*0.232*			0.003*			0.001*
Alive	87	42	45		28	59		48	39		45	42		40	47		50	37	
Died	12	4	8		9	3		2	10		4	8		11	1		1	11	

*FOXP3* Forkhead box P3, *TNC* triple-negative cancer, *N/A* not applicable,* i* intratumoral,* s* stromal, *TILs* Tumor-infiltrating lymphocytes

*P value is significant

*χ2 test

### Univariate and multivariate Cox regression analysis correlation with CD4, CD8, and FOXP3 levels with RFS and OS

Univariate and multivariate Cox regression analysis of RFS and OS were performed using clinicopathological findings and expression of TILs (Tables 2, 3). Univariate analysis revealed that the conventional clinicohistological tumor parameters, including age, tumor size, histological grade, lymph node status, and TILs were not prognostically significant. Furthermore, the iCD4 + TILs demonstrated a significant association with RFS (*p* = 0.044), but no significant difference in terms of OS (*p* = 0.074). Both in the RFS and OS, sCD4 + TILs patients showed no significance in univariate analysis (*p* = 0.261; *p* = 0.254). However, the expressions of sCD8 + and sFOXP3 + TILs were associated with RFS and OS (*p* = 0.020; *p* = 0.032). The iCD4 + , sCD8 + and sFOXP3 + TILs found to have significant prognostic value in univariate analysis were selected for Cox proportional hazard analyses and the significance of their prognostic association was confirmed by multivariate assessment. In multivariate analyses, patients with high expression of iCD4 + TILs had a significantly longer RFS (HR 0.172, 95% CI 0.037–0.792, *p* = 0.024). Increased infiltration of sCD8 + TILs was found to be a favorable prognostic factor in RFS (HR 0.225, 95% CI 0.061–0.836, *p* = 0.026) and OS (HR 0.263, 95% CI 0.071–0.975, *p* = 0.046). In contrast, a low sFOXP3 + TILs level was found to be significantly associated with favorable RFS (HR 7.426, 95% CI 1.596–34.552, *p* < 0.011) and OS (HR 5.467, 95% CI 1.192–25.07, *p* = 0.029) (Table 4).

**Table 3:** Hazards for triple-negative cancer (TNC) relapse-free survival (RFS) in the entire cohort with univariate and multivariate analyses

Clinicopathological feature	Univariate analysis			Multivariate analysis			
	HR	95.0% CI	*P *value*	HR	95.0% CI	*P *value*	
Age (< 60 vs. ≥ 60)	0.323	0.087–1.193	*0.090*				
Tumor size (2 cm vs. > 2 cm)	2.527	0.807–7.915	*0.111*				
Histological grade (I, II vs. III)	2.149	0.520–8.871	*0.290*				
Lymph node status (absent vs. present)	1.678	0.815–3.455	*0.160*				
iTILs (high vs. low)	0.584	0.185–1.846	*0.360*				
sTILs (high vs. low)	0.499	0.150–1.659	*0.257*				
iCD4 (low vs. high)	0.210	0.046–0.959	0.044*	0.172	0.037–0.792	0.024*	
sCD4 (low vs. high)	1.992	0.599–6.626	*0.261*				
iCD8 (low vs. high)	0.303	0.0082–1.119	*0.073*				
sCD8 (low vs. high)	0.213	0.058–0.785	0.020*	0.225	0.061–0.836	0.026*	
iFOXP3 (low vs. high)	0.333	0.090–1.230	*0.099*				
sFOXP3 (low vs. high)	5.804	1.265–26.62	0.024*	7.426	1.596–34.552	0.011*	
Ratio of immune cells							
iCD4/CD8	0.369	0.100–1.365	*0.135*				
sCD4/CD8	1.362	1.049–1.769	0.021*	1.003	0.650–1.549	*0.988*	
iCD8/FOXP3	0.626	0.199–1.973	*0.424*				
sCD8/FOXP3	0.200	0.065–0.616	0.005*	0.130	0.025–0.669	0.015*	
iFOXP3/CD4	0.356	0.096–1.314	*0.121*				
sFOXP3/CD4	1.858	1.160–2.977	0.010*	2.766	1.443–5.302	0.002*	

Multivariate cox regression analyses were performed for all potential variables that were significantly associated with survival in univariate analysis *RFS* recurrence-free survival. *TNC* triple-negative cancer, *HR* hazard ratio, *CI* confidence interval, i intratumoral, s stromal, *TILs* tumor-infiltrating

lymphocytes, *FOXP3* Forkhead box P3

^*^P value is significant

**Table 4:** Hazards for triple-negative cancer (TNC) overall survival (OS) in the entire cohort with univariate and multivariate analyses

Clinicopathological feature	HR	Univariate analysis	*P *value*	HR	Multivariate analysis	*P *value*	
		95.0% CI			95.0% CI		
Age (< 60 vs. ≥ 60)	0.316	0.08501.169	*0.084*				
Tumor size (2 cm vs. > 2 cm)	3.012	0.962–9.431	*0.058*				
Histological grade (I, II vs. III)	2.161	0.472–9.886	*0.321*				
Lymph node status (absent vs. present)	1.820	0.868–1.169	*0.113*				
TILs (high vs. low)	0.476	0.143–1.585	*0.226*				
iCD4 (low vs. high)	0.231	0.051–1.055	*0.059*				
sCD4 (low vs. high)	2.014	0.604–6.712	*0.254*				
iCD8 (low vs. high)	0.304	0.082–1.124	*0.074*				
sCD8 (low vs. high)	0.239	0.065–0.885	0.032*	0.263	0.071–0.975	0.046*	
iFOXP3 (low vs. high)	0.305	0.082–1.133	*0.076*				
sFOXP3 (low vs. high)	5.944	1.298–27.22	0.022*	5.467	1.192–25.07	0.029*	
Ratio of immune cells							
iCD4/CD8	0.413	0.111–1.530	*0.186*				
sCD4/CD8	1.368	1.042–1.797	0.024*	1.059	0.673–1.665	*0.804*	
iCD8/FOXP3	0.619	0.196–1.953	*0.414*				
sCD8/FOXP3	0.215	0.070–0.661	0.007*	0.157	0.031–0.797	0.026*	
iFOXP3/CD4	0.354	0.096–1.307	*0.119*				
sFOXP3/CD4	2.224	1.347–3.669	0.002*	3.386	1.684–6.807	0.001*	

Multivariate cox regression analyses were performed for all potential variables that were significantly associated with survival in univariate analysis *OS* overall survival. *TNC* triple-negative cancer, *HR* hazard ratio, *CI* confidence interval, i intratumoral, s stromal, *TILs* tumor-infiltrating lymphocytes, *FOXP3* Forkhead box P3

^*^The *P* value is significant

Moreover, the sCD4/CD8, sCD8/FOXP3, and sFOXP3/CD4 ratios were significantly associated with both RFS and OS in univariate analysis. We investigated these variables for their independent association with RFS and OS using a multivariate Cox regression model. The results revealed that sCD8/FOXP3 had prognostic significance for RFS (HR 0.130, 95% CI 0.025–0.669, *p* = 0.015) and OS (HR 0.157, 95% CI 0.031–0.797, *p* = 0.026). The sFOXP3/CD4 ratio was also significantly associated with RFS (HR 2.766, 95% CI 1.443–5.302, *p* = 0.002) and OS (HR 3.386, 95% CI 1.684–6.807, *p* = 0.001).

**Fig. 2:**
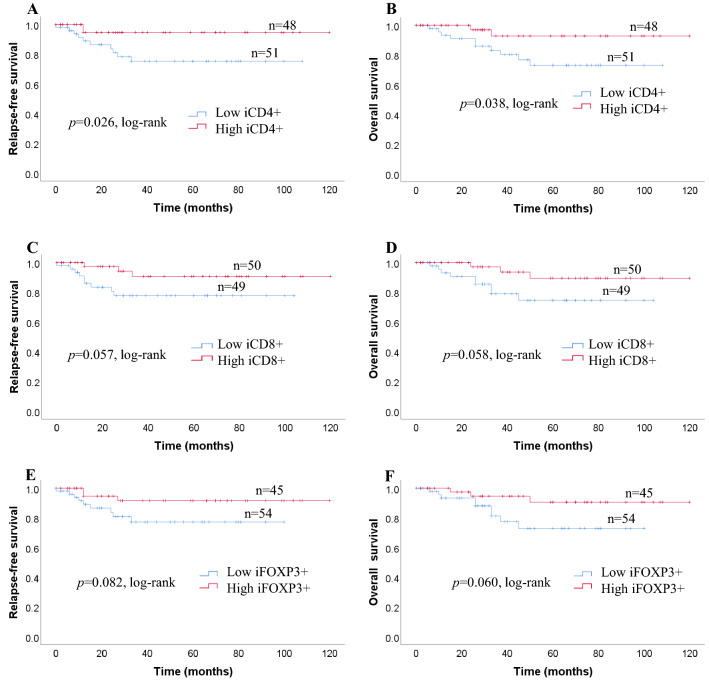

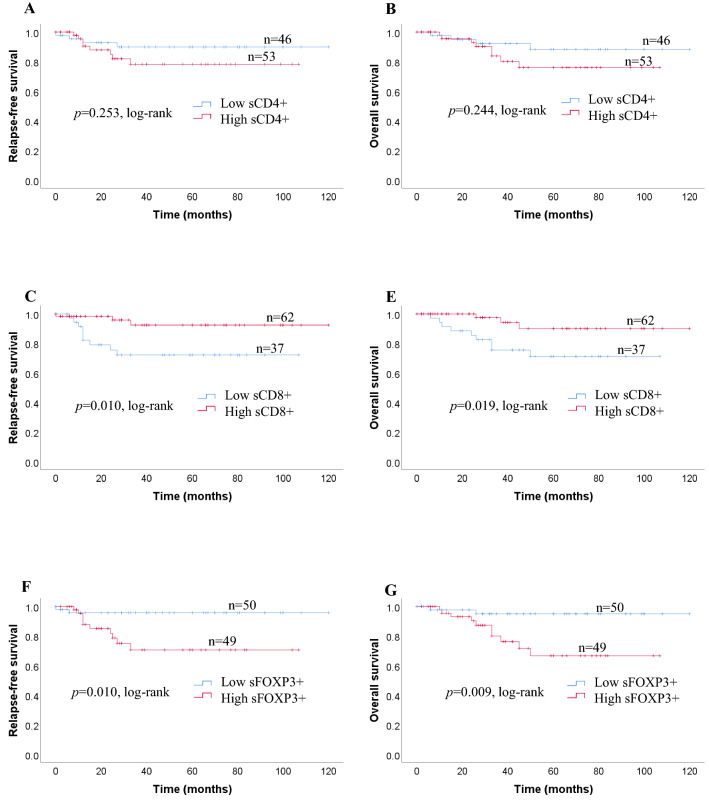

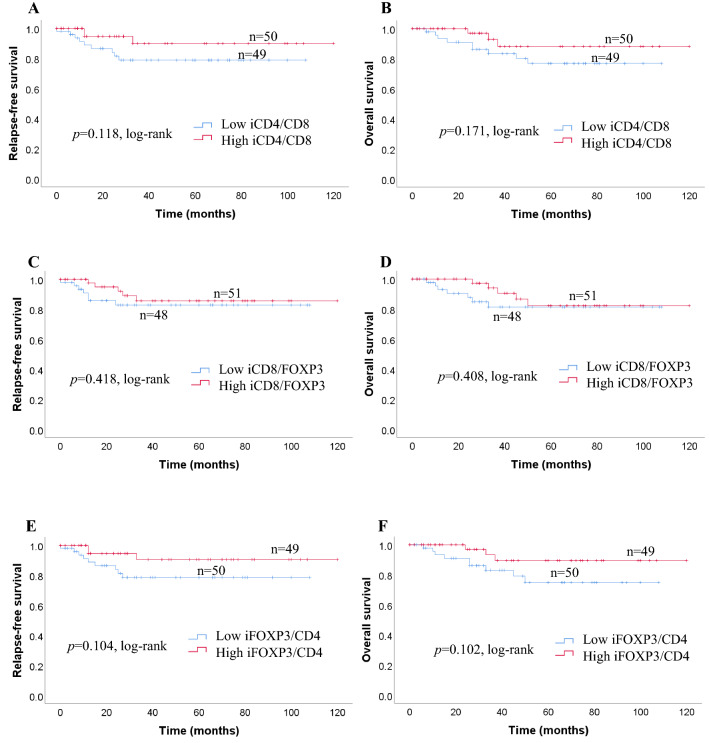

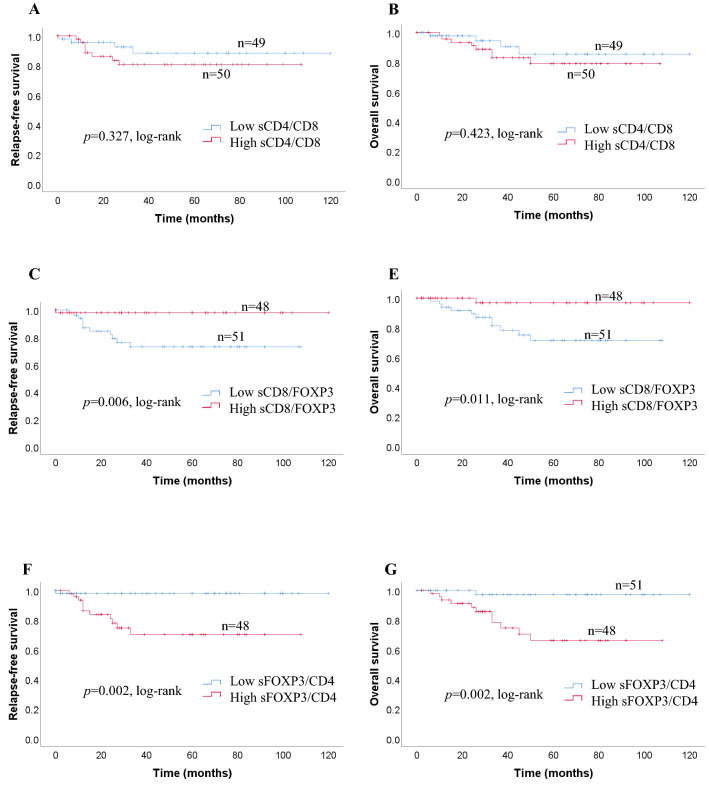
**a** Recurrence-free survival (RFS) and overall survival (OS) in patients with iCD4 + TILs, iCD8 + TILs, and iFOXP3 + TILs. Estimated Kaplan–Meier curves of RFS (**a**) and OS (**b**) in patients with high or low iCD4 + TILs, those of RFS (**c**) and OS (**d**) in patients with high or low iCD8 + TILs, and those of RFS (**e**) and OS (**f**) in patients with high or low iFOXP3 + TILs. **b** Prognostic significance of lymphocytic variables in breast cancer. Kaplan–Meier curves for overall survival (OS) and relapse-free survival (RFS) were stratified by the median values as the cut-off for prognostic evaluation and divided into low or high lymphocytic variable subsets. The blue solid line indicates patients with low values and the red solid line high values. sCD4 + TILs did not demonstrate prognostic significance for RFS (**a**) and OS (**b**), but high sCD8 + TILs was associated with both prolonged RFS (**c**) and OS (**d**). In contrast, high sFOXP3 + TILs was associated with both reduced RFS (**e**) and OS (**f**). **c** Kaplan–Meier survival curves illustrating the relapse-free survival (RFS) and overall survival (OS) according to the ratio of iCD4/CD8 (**a**, **b**), iCD8/FOXP3 (**c**, **d**) and iFOXP3/CD4 (**e**, **f**). **d** Recurrence-free survival (RFS) and overall survival (OS) in patients with different sCD4/CD8, sCD8/FOXP3, and sFOXP3/CD4 ratios. Estimated Kaplan–Meier curves of RFS (**a**) and OS (**b**) in patients with high or low sCD4/CD8 ratios, those of RFS (**c**) and OS (**d**) in patients with high or low sCD8/FOXP3 ratios, and those of RFS (**e**) and OS (**f**) in patients with high or low sFOXP3/CD4 ratios

We investigated survival with regard to the different expressions of CD4 + TILs, CD8 + TILs FOXP3 + TILs status using the Kaplan–Meier method and log-rank test. Patients with high expression of iCD4 + TILs had significantly longer RFS (*p* = 0.026) and OS (*p* = 0.038) than those with low expressions of iCD4 + TILs (Fig. 2a). The expressions of iCD8 + TILs, and iFOXP3 + TILs, were not related to either RFS (*p* = 0.057, *p* = 0.082) or OS (*p* = 0.058, *p* = 0.060, respectively; Fig. 2a). sCD4 + TILs were not significantly correlated with OS (*p* = 0.244) or RFS (*p* = 0.253) in patients with TNC (Fig. 2b). In contrast, a high number of sCD8+ and low number of sFOXP3 + TILs were significantly correlated with favorable RFS (*p* = 0.010; *p* = 0.010) and OS (*p* = 0.019; *p* = 0.009, respectively; Fig. 2b). Kaplan–Meier analysis revealed survival differences based on the ratio between *i* and *s* infiltration of immune cells (CD4/CD8, CD8/FOXP3, FOXP3/CD4). We observed no significant difference in the ratios of *i* immune cells (CD4/CD8, CD8/FOXP3, FOXP3/CD4) between RFS (*p* = 0.118, *p* = 0.418, *p* = 0.104) and OS (*p* = 0.171, *p* = 0.408, *p* = 0.102) (Fig. 2c). With regard to the ratio of immune cells, no significant association was seen between the sCD4/CD8 ratio and RFS or OS (*p* = 0.327; *p* = 0.423). Patients with greater changes in the sCD8/FOXP3 ratio had significantly better RFS and OS compared with those with smaller changes (*p* = 0.006; *p* = 0.011) (Fig. 2d). Furthermore, we found that patients with a high sFOXP3/CD4 ratio had a significantly poorer RFS and OS (*p* = 0.002; *p* = 0.002) (Fig. 2d).

## Discussion

The characteristic features of TNC are large anaplastic cells and poor prognosis. Invasive carcinoma including medullary features with massive TILs has a better prognosis than the typical types of invasive mammary carcinomas [3, 23]. Furthermore, a recent report suggested the prognostic importance of TILs in high-grade breast cancers. Kurozumi et al. recently investigated the relationship between TILs and prognosis in 294 cases and reported that high stromal TILs expression was a good prognostic marker in ER-negative cancers [24]. Ibrahim et al. also demonstrated that TILs were significantly correlated with a favorable breast cancer outcome in ER-negative tumors using meta-analysis including data on 2,987 patients [25]. However, we could not find significant differences in either iTILs or sTILs regarding the prognosis in TNC. Therefore, it is difficult to reach a conclusion regarding the prognosis of breast cancer based only on the TILs in TNC. Our results suggest that this prognosis in TNC is due, at least in part, to the presence of immune cell types that are closely associated with the tumor.

We found that TNC patients with a good prognosis had a predominance of sCD8 + TILs in both RFS and OS. Lymphocytes infiltrating a tumor indicate a local immune response and they play an important role in tumor progression [11, 12, 26]. The majority of infiltrating lymphocytes in tumors are CD8 + TILs and these have a cytotoxic effect [12, 27]. In several organs, high levels of CD8 + TILs infiltration were associated with better prognosis [5–10]. In breast cancer, Ali et al. reported that iCD8 + and sCD8 + T cell infiltration was also associated with a significant reduction in the relative risk of death [11]. Furthermore, Liu et al. reported that iCD8 + and sCD8 + tumor-infiltrating lymphocytes are an independent prognostic factor associated with better survival in TNC [28]. Therefore, the greater predominance of CD8 + lymphocyte infiltration in TNC suggests that a strong immune response is occurring. However, we found increased infiltration of iCD4 + TILs was significantly associated with good prognosis only in RFS by multivariate analysis. In contrast, there have been a few reports examining the role of CD4 + TILs in breast cancers; they were associated with more aggressive behavior. Huang et al. reported that iCD4 + TILs negatively correlated with RFS in breast cancer [26]. Rubbert et al. reported a predominance of sCD4 + TILs among tumor-infiltrating lymphocytes in patients with larger tumors [29]. Furthermore, Macchetti et al. observed that in patients with lymph node metastasis, there was increased infiltration of sCD4 + TILs with a corresponding reduction in CD8 + cells [30]. Since CD4 + TILs are expressed in many T cell subsets including T helper 1 (Th1) cells, T helper 2 (Th2) cells and Tregs, each of these may have a different impact on prognosis. Th1 cells secrete several cytokines such as interferon gamma (IFNg), transforming growth factor beta (TGFβ), tumor necrosis factor alpha (TNF), and interleukin 2 (IL-2) [31]. These cytokines are involved in the function of CD8 + TILs and protect against tumor development and progression. In contrast, Th2 cells express several types of interleukin and induce loss of cytotoxicity [32]. Thus, CD4 + TILs that include many T cell subsets may explain why iCD4 + TILs was different in TNC.

In the present study, TNC patients with a good prognosis showed significantly lower expression of sFOXP3 + Tregs. Tregs are important mediators of immune tolerance that suppress T cell effects and inhibit immune-mediated tissue damage. FOXP3 is a member of the forkhead/winged-helix family of transcription factors related to the regulation of the development and function of the immune system. Excess FOXP3 expression leads to Treg proliferation and severe immunodeficiency, whereas lack of FOXP3 results in immune system activation and aggressive lymphoproliferation [15, 16, 33]. FOXP3-expressing Tregs are reported to be abundant in tumor infiltrates and are involved in the immune escape mechanisms promoted by cancer. In several types of cancer, high levels of Tregs infiltration around the tumor were found to be correlated with poor prognosis [27, 34]. However, opinions vary among researchers regarding the role of FOXP3 + in breast cancer. Castaneda et al. evaluated 98 TNC patients and higher expression of sFOXP3 + Tregs in TILs showed longer disease-free survival [35]. However, one limitation of this study is that they did not perform univariate and multivariate Cox regression analysis. In contrast, Kim et al. reported that higher numbers of FOXP3-expressing Tregs were associated with shorter RFS in breast cancers [36]. Furthermore, Peng et al. reported that high grade infiltrating ductal carcinoma with good prognosis showed significantly lower expression of FOXP3 [37]. In addition, a decreased ratio of CD8 + TILs to FOXP3 + Tregs infiltrating and surrounding tumors correlated with poor prognosis in breast cancer [38]. Thus our results are consistent with these findings that TNC patients with a good prognosis have lower expression of sFOXP3 + Tregs.

We also investigated the CD4/CD8, CD8/FOXP3, and FOXP3/CD4 ratio because there have been several studies that reported the CD8/FOXP3 ratio in breast cancer. Liu et al. reported an increased ratio of CD8/FOXP3 in the peritumoral area of non-luminal carcinoma and indicated good survival of breast cancer [27]. A recent study by Miyashita et al. demonstrated that a high sCD8/FOXP3 ratio was associated with improved prognosis in TNC [39]. Furthermore, our study confirmed that not only sCD8/FOXP3, but also the sFOXP3/CD4 ratio, were significantly associated with both RFS and OS. It seemed that both FOXP3 and CD4 were associated with tumor progression, but FOXP3 was a stronger indicator. These results suggest that activation of cytotoxic TILs and Tregs may affect the clinical outcome.

## Conclusion

The present study demonstrated no difference in either iTILs or sTILs and survival in TNC. However, we found higher numbers of iCD4 + TILs were significantly associated with good prognosis in RFS. Further, decreased sFOXP3 + TILs infiltrate and higher numbers of sCD8 + TILs in TNC were associated with a significantly good prognosis in both RFS and OS. Therefore, we should not simply focus on the TILs level in TNC. It is possible that a local immune response leading to killer cell expression occurs in some cases and suppression by regulating Tregs occurs in other cases. The difference in clinical outcome of TNC may be due to the subtype of the infiltrating TILs.
